# Postoperative Rehabilitation Protocol Following Arthroscopic Rotator Cuff Repair: A Prospective Single-Arm Pragmatic Interventional Study

**DOI:** 10.3390/medicina58060729

**Published:** 2022-05-28

**Authors:** Hyunjoong Kim, Seungwon Lee

**Affiliations:** 1 Department of Physical Therapy, Graduate School, Sahmyook University, 815, Hwarang-ro, Seoul 01795, Korea; doong18324@gmail.com; 2 Department of Physical Therapy, Sahmyook University, 815, Hwarang-ro, Seoul 01795, Korea

**Keywords:** shoulder pain, rotator cuff injuries, arthroscopic surgery, manual therapy, rehabilitation exercise

## Abstract

*Background and Objectives*: Rotator cuff tear is the most common cause of shoulder pain. If nonsurgical treatment fails, arthroscopic rotator cuff repair (ARCR) is recommended. Since the standards for rehabilitation after ARCR are not clear, various rehabilitation methods have been suggested. This study intends to investigate the effect on the recovery phase of ARCR patients through a postoperative rehabilitation protocol (PRP) that considers the healing process and rehabilitation trend. *Materials and Methods*: This single-arm, pragmatic intervention study was conducted on 30 patients, two weeks postoperative day (POD) after ARCR. ARCR patients received intervention for six weeks from POD two-week, and pain intensity and shoulder function were evaluated at two-week intervals until POD 12-week, and range of motion (ROM) was evaluated at POD four-week and eight-week. *Results*: In this study, all variables improved over time (*p* < 0.05). As a result of the comparison between time points, a significant improvement was found in shoulder function at POD 6-week. In addition, the effect size had a large effect on ROM (flexion, scaption flexion, abduction, and external rotation) and shoulder function. *Conclusions*: Management through the PRP based on scientific evidence in the strategy of postoperative rehabilitation of patients with ARCR is effective for pain intensity, ROM, and shoulder function.

## 1. Introduction

Shoulder pain accounts for 7–26% of the total population [1]. The most common cause of shoulder pain is rotator cuff tears [2]. According to the clinical practice guidelines of the American Academy of Orthopedic Surgeons (AAOS), physical therapy in non-operative management can lead to clinical improvement, but it has been reported that rotator cuff tears progress over time [3]. Therefore, when nonsurgical management of rotator cuff tears fails, arthroscopic rotator cuff repair (ARCR) is recommended surgically [1,4].

The goal of rehabilitation following ARCR is to normalize the biomechanics and function of the shoulder complex by promoting the recovery of muscles and tendons and balancing the movements of the scapula and humerus [5]. However, the postoperative rehabilitation protocol is controversial, and there are no standardized criteria for the immobilization period and range of motion (ROM) [6]. In many studies, joint immobilization is generally performed with a sling for four to six weeks, with a total rehabilitation period of 4 to 12 months [4,7,8].

Physical therapy interventions, such as manual therapy and exercise, are the main methods of nonsurgical management of the rotator cuff. This aims to correct by removing the factors that contribute to pain and dysfunction rather than treating specific pathological factors [7]. Similarly, physical therapy intervention in post-ARCR management is an important factor for a clinically positive prognosis.

However, a conclusive therapeutic basis for various physiotherapy interventions has not been identified [8,9,10]. This means that the application of an appropriate protocol considering factors that may affect the healing process and prognosis is required [11]. A relatively recent large-scale study confirmed that joint stiffness decreased, muscle strength increased in passive and active range of motion, and muscle strengthening exercises [12].

Therefore, this study aimed to investigate the effect on pain intensity, ROM, and shoulder function through a postoperative rehabilitation protocol (PRP) following rotator cuff repair.

## 2. Materials and Methods

### 2.1. Study Design

This study was designed according to the Strengthening the Reporting of Observational Studies in Epidemiology (STROBE) guidelines as a prospective, open-label, single-group, interventional study. The study was conducted from March to September 2021, and the protocol was registered on 13 January 2021 (ClinicalTrial.gov (accessed on 19 April 2022): NCT04711616).

### 2.2. Participants and Ethics

This study included patients admitted to The Better Hospital for postoperative rehabilitation after ARCR. Participants were recruited voluntarily through a sports rehabilitation center bulletin board. The eligibility assessment was conducted according to the inclusion and exclusion criteria [13]. The inclusion criteria were adults aged ≥ 18 years, 2 weeks after ARCR, and who wanted to participate in the study. The exclusion criteria were as follows: age ≥ 65 years, additional augmentation, history of surgery, and osteoarthritis of the shoulder joint.

This study was approved by the Institutional Review Board of Sahmyook University (No. 2-1040781-A-N-012021012HR). Informed consent was obtained from all participants before the start of the study.

### 2.3. Sample Size

Population estimation using standard deviation with a single group design was performed using a power calculator (Version 7.12, Institut Municipal d’Investigació Mèdica, Barcelona, Spain). It was calculated using the standard deviation (1.3) at the time point when a significant change in pain appeared in the early mobilization group [1]. A sample size of 26 participants randomly selected will suffice to estimate with a 95% confidence and a precision +/− 0.5 units, a population mean of a value that has been considered present a standard deviation of 1.3 units. It has been anticipated a replacement rate of 0%. Thirty participants were recruited for statistical accuracy and central limit theorem.

### 2.4. Postoperative Operative Rehabilitation Protocol

Postoperative rehabilitations, according to the healing process [14] in PRP following ARCR, were described by Van Der Meijden et al. [15] and Jung et al. [16]. In addition, based on the study by Berton et al. [11], it was divided into a maximal protection phase and a minimal protection active phase.

#### 2.4.1. Physical Agents

Physical agents consisted of superficial heat therapy, microwave therapy, and transcutaneous electrical nerve stimulation (TENS), five sessions per week for 6 weeks, 35 min per session (Table 1) [17]. For superficial heat therapy, infrared radiation (IR) by IR-2014 (AJINMEDICAL, Jeonju, Korea) was used to promote surface circulation [18]. Participants received IR for 15 min in a sitting position. For microwave therapy, Biowave HM-801 (Hanil-TM, Seoul, Korea) was used for deep tissue circulation [19]. After the IR was completed, it was applied for 5 min at a distance of 20 cm from the biowave in a sitting position. For TENS, pain control was set to an automatic modulation intense TENS of 100–300 Hz [20]. After IR and biowave, BM-420 (Hanil-TM, Seoul, Korea) was used and applied for 15 min.

#### 2.4.2. Manual Therapy

Manual therapy was divided into soft tissue mobilization for 20 min and joint mobilization for 10 min and was performed 5 times a week for 6 weeks. Soft tissue mobilization was applied to the upper extremities and peri-scapular muscles because it effectively improved the pain and function of impingement syndrome [21]. Joint mobilization was applied to the glenohumeral joint, scapular, and thoracic spine based on the fact that it was effective for posteroinferior capsular tightness and decreased cervicothoracic extension in rotator cuff tendinopathy [22] (Table 1).

#### 2.4.3. Exercise

The exercise was divided into simple ROM exercises and therapeutic exercises, and the intensity of the exercise was tailored to the healing process [11]. For continuous passive motion (CPM) during ROM exercise, ARTUS-703S (Eugene Medicare, Seoul, Korea) was used to maintain mobility [23]. Therapeutic exercises focused on conscious exercise for scapular dyskinesis. Furthermore, a stretching exercise was added to consider upper cross syndrome [24,25,26] (Table 1).

### 2.5. Outcomes

Participants received six assessments at 2-week intervals during the 10-week study period. Pre-test before intervention (T0), mid-test twice (T1, 2), post-test after intervention (T3), and follow-up twice after discharge (T4, T5). Only shoulder ROM was assessed by an assessor, meanwhile, all other outcome measures were assessed using self-report questionnaires.

#### 2.5.1. Primary Outcome Measures

The intensity of the pain was divided into the usual and worst pain groups. A numeric pain rating scale (NPRS) consisting of 0 points (no pain) to 10 points (most severe pain) was used [27]. Additionally, a score of about 5 was defined as pain that interferes with daily life. NPRS was a minimal clinically important difference (MCID) of 1.1–2.2 points [27,28].

#### 2.5.2. Secondary Outcome Measures

Active ROM was evaluated for shoulder joint ROM using a goniometer. ROM evaluation using the goniometer was reported to have high intraobserver reliability (intraclass correlation coefficient [ICC] = 0.91–0.99) [29]. The motions of the evaluated shoulder joints were flexion, scaption flexion, abduction, horizontal adduction, external rotation, and internal rotation [1].

Should function was analyzed using disabilities of the arm, shoulder, and hand (DASH), shoulder pain and disability index (SPADI), and a simple shoulder test (SST). The DASH has 30 items on a 5-point scale per item, and the reported MCID ranges from 8.1 to 13 [30,31]. The SPADI consists of two subitems (pain and disability) and consists of 13 items. For SPADI, the reported MCID scores ranged from 14.1 to 20.6% [30]. The SST consists of 12 items and selects “yes” or “no”. The reported MCID of SST was 2 points [32]. For all three self-reported questionnaires that evaluated shoulder function, higher scores indicated shoulder dysfunction.

### 2.6. Data Analysis

All statistical analyses were performed using SPSS (version 25.0, IBM Corp., Armonk, NY, USA). The general characteristics of the participants were expressed using descriptive statistics. For each evaluated variable, repeated measures analysis of variance was used to analyze the change over time. It is expressed as partial eta squared ($\eta_{p}^{2}$) (small, 0.01; moderate, 0.06; large, 0.14) [33] for quantification through effect size. If there was a change with time, it was analyzed using Bonferroni’s method to compare the change between time points. ROM, which was evaluated only twice, did not satisfy the normal distribution; therefore, it was analyzed using the Wilcoxon signed-rank test, and the effect size was expressed by Cohen’s d (small, 0.20; moderate, 0.5; and large, 0.80) [34]. All statistical significance levels were set at *p* < 0.05.

## 3. Results

**Figure 1:**
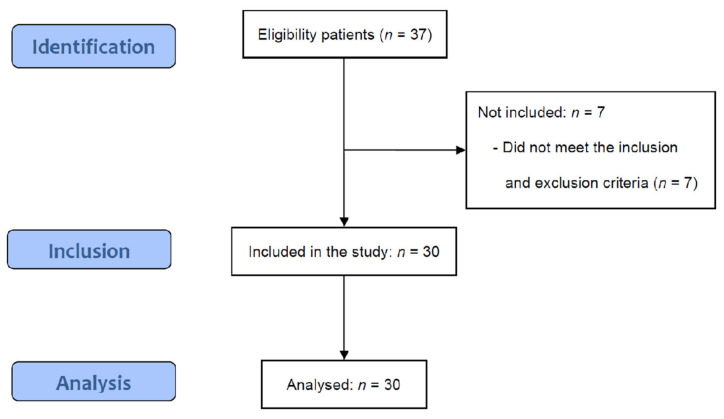
STROBE flow diagram.

Figure 1 is a flow diagram of this study based on the STROBE guidelines. A total of 37 potential participants were screened for eligibility and seven participants were excluded. Finally, 30 enrolled participants were assessed at two-week intervals from postoperative day (POD) 2 weeks to POD 12 weeks.

### 3.1. Characteristics of the Enrolled Participants

Table 2 shows the general characteristics of the participants. The ratio of females (63.33%) to the affected right side (70.00%) was high. Details of the ARCR that the participants underwent included repair of the supraspinatus and/or subscapularis, capsular release, biceps tenodesis, and subacromial decompression. Capsular release, biceps tenodesis, and subacromial decompression were additionally performed as needed. The procedure for ARCR is as follows. View the tear through the scope and create a portal in the skin for instrument insertion. It was passed across the tear using a suture passer and tied to the tendon. Next, suture anchors were inserted and anchored to the bone. When the suture attached to the anchor was exposed, the tendon was tied and fixed [35,36].

### 3.2. Change in Pain Intensity

**Figure 2:**
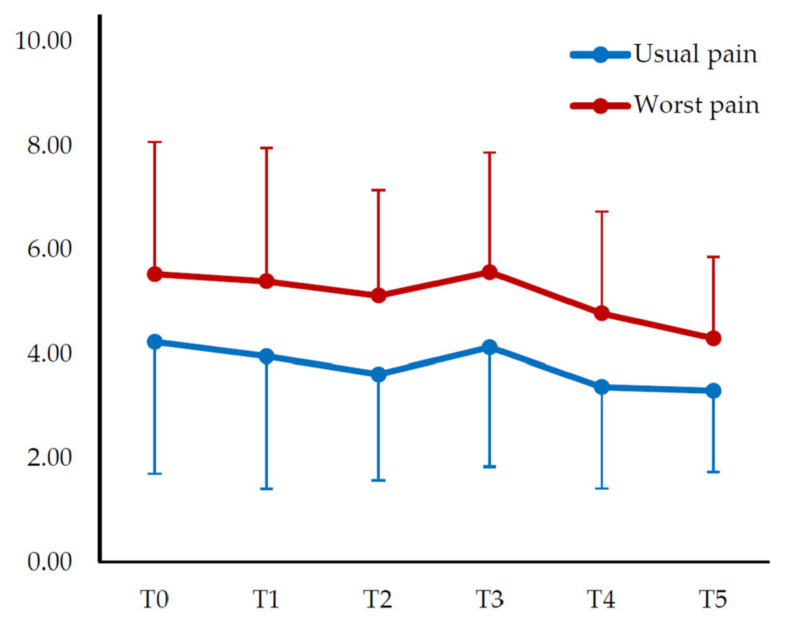
Change in pain intensity over time. Values are expressed as mean and standard deviation (error bars).

Similar results were obtained for both the usual and worst pain. There was a change with time (F = 3.731; $\eta_{p}^{2}$ = 0.114), but there was no significant difference between the time points (*p* > 0.05). Similarly, there was a change with time in the worst pain (F = 3.572; $\eta_{p}^{2}$ = 0.110), but there was no significant difference between time points (*p* > 0.05) (Table 3) (Figure 2).

### 3.3. Change in Range of Motion

For ROM, only two evaluations (T1 and T3) were performed. A significant improvement was observed in all motions (*p* < 0.01), and in particular, the effect size in motion, except for horizontal adduction and internal rotation, had a large effect (d > 0.80) (Table 4).

### 3.4. Change in Shoulder Function

**Figure 3:**
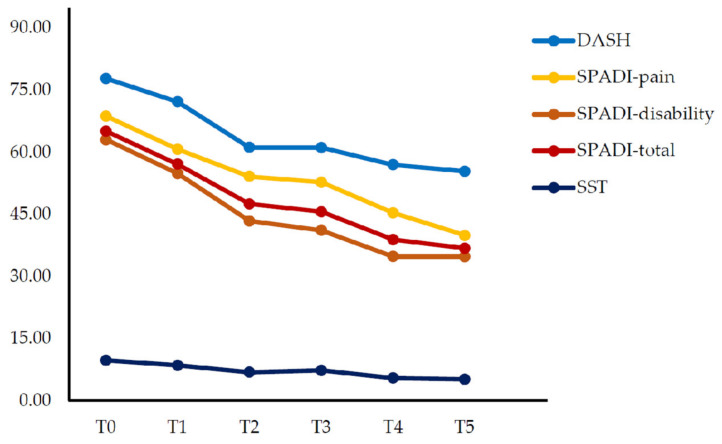
Changes in shoulder function over time. Values are expressed as mean.

In shoulder function, DASH, SPADI, and SST showed significant improvement over time (*p* < 0.001). In DASH, the effect size was found to have a large effect ($\eta_{p}^{2}$ = 0.479), and there was a significant difference between T2, T3, T4, and T5 compared with T0 (*p* < 0.001). In SPADI pain, the effect size was found to have a significant effect ($\eta_{p}^{2}$ = 0.433), and there was a significant difference between T2, T3, T4, and T5 compared with T0 (*p* < 0.01). In SPADI-disability, the effect size had a large effect ($\eta_{p}^{2}$ = 0.417), and there was a significant difference between T2, T3, T4, and T5 compared with T0 (*p* < 0.001). In the SPADI-total, the effect size was found to have a large effect ($\eta_{p}^{2}$ = 0.462), and there was a significant difference between T2, T3, T4, and T5 compared with T0 (*p* < 0.001). In SST, the effect size had a large effect ($\eta_{p}^{2}$ = 0.451), and there was a significant difference between T2, T3, T4, and T5 compared with T0 (*p* < 0.05) (Table 3) (Figure 3).

## 4. Discussion

This interventional study evaluated pain intensity, ROM, and shoulder function to investigate the effect of a PRP tailored to the healing process in rehabilitation after ARCR. In addition, it is a pragmatic trial to confirm the effectiveness of the rehabilitation protocol performed in an actual rehabilitation hospital. In this study, there was a significant improvement in all measured variables (*p* < 0.05). When comparing the effect size based on the statistical method, ROM (flexion, scaption flexion, abduction, and external rotation) and shoulder function (DASH, SPADI, and SST) had a large effect ($\eta_{p}^{2}$ > 0.14; d > 0.80).

Regarding pain intensity, a primary outcome measure, both usual pain and worst pain showed significant improvement (F = 3.731; F = 3.572), but no difference between time points (*p* > 0.05), and the effect size also showed a moderate effect. In MCID (1.1–2.2 points), the usual pain did not show a significant decrease, but there was a significant decrease of 1.24 points in the worst pain. In the study by Duzgun, et al. [37], activity pain was measured using a visual analog scale, and the results of the accelerated protocol and the slow protocol from 1-week to 12-week of POD were similar to the trend of pain reduction in this study. Moreover, in a comparative study of 105 patients with ARCR with or without early passive ROM exercise, there was no significant difference in the preoperative level at 3, 6, and 12 months after ARCR [38]. Therefore, to date, there is a limit to pain control in rehabilitation after surgery. However, in the results, where the phase of the protocol is presented, the slight change in shoulder function along with the increase in pain between T2 and T3 is the time when the active ROM starts, and the intensity is increased one step, so it is confirmed that it is a suitable result according to the protocol. The strength of this study is that shoulder function increases with pain control after the minimal protection active phase.

In the results of shoulder ROM, a significant improvement was found in all motions (*p* < 0.01). These results were similar to those of subacromial corticosteroid injection after ARCR in the study by Ha, Kim and Kim [38] at three months, but the increase in external rotation and internal rotation was higher in this study. In a review of the rehabilitation concept after ARCR, it was reported that the goal of the intermediate stage of postoperative rehabilitation (POD 7-week to 12-week) is to recover complete active ROM [39]. Therefore, depending on the rehabilitation phase, the earlier the full ROM, the better the prognosis. The full range of shoulder motion was approached at POD 8-week, so it was an effective protocol to increase ROM.

As a result of shoulder function, DASH, SPADI, and SST all showed changes over time (*p* < 0.001). In the comparison between time points, a significant improvement was observed between POD six-week and POD two-week (*p* < 0.01). In addition, it showed a difference of approximately twice as high as the previously reported MCID of DASH, SPADI, and SST. In the Duzgun, Baltaci and ATAY [37] study comparing programs according to the rehabilitation period, the DASH score showed significant improvement up to 8, 12, and 16 weeks in the accelerated rehabilitation protocol, similar to this PRP. In addition, all participants enrolled in this study underwent subacromial decompression. In a comparative study, according to the presence or absence of subacromial decompression in patients with ARCR, there was no significant difference in the results after two years, but it was reported that the group that received subacromial decompression had a higher DASH [40]. These results were similar to those of this study, evaluated up to POD 12-week, which all showed positive functional improvement.

Although this was a prospective study, there was a limitation in the interpretation of the results due to the vulnerability to bias with a single-group design. Furthermore, although it is a longitudinal design, the long-term effect is unknown because the entire study period was 10 weeks. However, it can be said to be pragmatic as a rehabilitation protocol implemented in a real clinical setting. Furthermore, it would have been more beneficial if comparisons with historical groups were added. As in this study, various protocols are still proposed for postoperative rehabilitation following ARCR, so the prognosis that can be predicted by comparing the results with long-term follow-up studies will be positive. Further studies recommend a randomized controlled trial using PRP.

## 5. Conclusions

This trial confirmed pain control, increased ROM, and improvement in shoulder function in participants with postoperative rehabilitation after ARCR consisting of six weeks of physical therapy intervention. In particular, large and significant changes in shoulder function questionnaires, along with a high increase in ROM, suggest that rehabilitation had a positive effect on patients’ daily life and participation in leisure activities.

## Figures and Tables

**Table 1:** Postoperative rehabilitation protocol.

Types	Component	Description	Dosage
Physical agents	Superficial heat therapy	In side-lying position, using infrared radiation	15 min per session 5 sessions per week
	Microwave therapy	In side-lying position, using biowave, at a distance of 20 cm	5 min per session 5 sessions per week
	TENS	In side-lying position, automatic modulation intense TENS of 100–300 Hz	15 min per session 5 sessions per week
Manual therapy	Soft tissue mobilization	Upper extremity and periscapular regions, in supine and side-lying positions, respectively	20 min per session 5 sessions per week
	Joint mobilization	Glenohumeral joint, scapular, and thoracic spine, in supine and side-lying positions, respectively	10 min per session 5 sessions per week
Exercises	*Maximal protection phase; Wearing shoulder abduction sling (POD 2-week to 4-week)*		
	ROM exercise	CPM: In a sitting position, the instrument was set to a scaption of 180° flexion to 20° of extension Active assisted ROM exercise: ROM exercise with correct movement under supervision	30 min per session 5 sessions per week
	Therapeutic exercise	Active exercise of the elbow and wrist, scapular conscious exercise, scapular setting exercise	20 min per session 5 sessions per week
	*Minimal protection active phase (POD 4-week to 8-week)*		
	ROM exercise	CPM: In a sitting position, the instrument was set to a scaption of 180° flexion to 20° of extension Active ROM exercise: ROM exercise with correct movement under supervision	30 min per session 5 sessions per week
	Therapeutic exercise	Scapular stabilization exercise, pectoralis and periscapular muscle stretching exercise (mild to moderate)	20 min per session 5 sessions per week

CPM, continuous passive motion; POD, postoperative day; ROM, range of motion; TENS, transcutaneous electrical nerve stimulation.

**Table 2:** Characteristics of enrolled participants.

Variables (*n* = 30)	Mean ± SD
Sex (male, %)	11 (36.67)
Affected side (left,%)	9 (30.00)
Age (years)	51.86 ± 4.76
Height (cm)	161.47 ± 6.06
Weight (kg)	63.20 ± 8.74
Body mass index (kg/m^2^)	23.78 ± 2.12
*Arthroscopic rotator cuff repair*	
Supraspinatus repair (*n*)	14
Subscapularis repair (*n*)	20
Capsular release (*n*)	14
Biceps tenodesis (*n*)	24
Subacromial decompression (*n*)	30

**Table 3:** Changes in pain intensity and shoulder function in postoperative rehabilitation.

Variables (*n* = 30)	T0 POD 2-Week	T1 POD 4-Week	T2 POD 6-Week	T3 POD 8-Week	T4 POD 10-Week	T5 POD 12-Week	Time F (*p*) ^(a)^	Effect Size ^(b)^
	Mean ± SD	Mean ± SD	Mean ± SD	Mean ± SD	Mean ± SD	Mean ± SD		
*Pain intensity*								
Usual pain	4.24 ± 1.55	3.97 ± 1.59	3.62 ± 1.86	4.14 ± 1.90	3.38 ± 1.88	3.31 ± 1.73	3.731 (0.013)	0.114
Worst pain	5.55 ± 2.53	5.41 ± 2.54	5.14 ± 2.03	5.59 ± 2.29	4.79 ± 1.95	4.31 ± 1.56	3.572 (0.011)	0.110
*Shoulder function*								
DASH	77.82 ± 19.51	72.18 ± 17.78	61.15 ± 16.33 ***	61.12 ± 17.90 ***	56.98 ± 16.58 ***	55.37 ± 17.69 ***	26.636 (0.000)	0.479
SPAD-pain	68.69 ± 22.94	60.69 ± 27.20	54.14 ± 23.46 **	52.76 ± 24.37 **	45.38 ± 23.71 ***	39.93 ± 23.49 ***	22.166 (0.000)	0.433
SPADI-disability	63.02 ± 23.09	54.87 ± 28.16	43.41 ± 23.26 ***	41.16 ± 25.34 ***	34.83 ± 22.83 ***	34.79 ± 25.39 ***	20.770 (0.000)	0.417
SPADI-total	65.10 ± 22.65	57.11 ± 27.53	47.53 ± 22.01 ***	45.62 ± 24.22 ***	38.89 ± 22.77 ***	36.81 ± 23.77 ***	24.900 (0.000)	0.462
SST	9.72 ± 2.96	8.55 ± 1.99	6.90 ± 1.68 ***	7.34 ± 3.05 *	5.48 ± 2.44 ***	5.17 ± 2.28 ***	23.801 (0.000)	0.451

^(a)^ Repeated measures analysis of variance, ^(b)^ Partial eta squared ($\eta_{p}^{2}$). DASH, disabilities of the arm, shoulder, and hand; POD, postoperative day; SD, standard deviation; SPADI, shoulder pain and disability index; SST, simple shoulder test. * *p* < 0.05, ** *p* < 0.01, *** *p* < 0.001, statistically significant difference from T0.

**Table 4:** Changes in the range of motion in postoperative rehabilitation.

Variables (*n* = 30)	T1	T3	Z	*p* ^(a)^	Effect Size ^(b)^
	Median	Median			
Flexion	162.50	180.00	−4.061	0.000	1.167
Scaption flexion	130.00	180.00	−4.013	0.000	1.355
Abduction	120.00	180.00	−3.449	0.001	0.907
Horizontala	110.00	130.00	−3.432	0.001	0.766
External rotation	60.00	80.00	−4.183	0.000	1.005
Internal rotation	50.00	50.00	−3.204	0.001	0.506

^(a)^ Wilcoxon signed rank test; ^(b)^ Cohen’s d.

## Data Availability

Not applicable.
